# Hallmarks of aging: middle-aging hypovascularity, tissue perfusion and nitric oxide perspective on healthspan

**DOI:** 10.3389/fragi.2024.1526230

**Published:** 2025-01-07

**Authors:** Teow J. Phua

**Affiliations:** Molecular Medicine, NSW Health Pathology, John Hunter Hospital, Newcastle, NSW, Australia

**Keywords:** aging hallmarks, geroscience, vascular aging, tissue perfusion, nitric oxide, healthspan, causal inference, triangulation of evidence

## Abstract

Aging is a complex process marked by various changes at both cellular and systemic levels, impacting the functioning and lifespan of organisms. Over time, researchers have pinpointed several significant hallmarks of aging that lead to the gradual deterioration of tissue function, regulation, and homeostasis associated with aging in humans. Despite this, the intricate interactions and cumulative effects of these hallmarks are still mostly uncharted territory. Understanding this complex web is a major challenge in Geroscience, yet it is crucial for developing effective strategies that promote healthy aging, reduce medical costs, and ensure the sustainability of health systems. Gaining insights in this area is essential for creating interventions that can slow the aging process, enhance healthspan, and decrease the likelihood of age-related diseases. The integration of knowledge from various fields concerning the middle-aging nitric oxide (NO)-mediated hypovascularity hypoxia hemodynamic hypothesis points to a systems-based approach to the biological hallmarks of aging. Key evidence suggests a systemic connection between the endocrine system (specifically sex hormones), endogenous NO deficiency, and the vascular system, which serves as a network of microvascular structures crucial for tissue perfusion functions at cellular level. These processes also involve oxidative stress and inflammation triggered by hypoxia.

## 1 Introduction

These are the 12 interconnected hallmarks of aging that encompass genomic instability, telomere shortening, epigenetic modifications, protein imbalance, impaired macro-autophagy, disrupted nutrient-sensing pathways, mitochondrial dysfunction, cellular senescence, stem cell exhaustion, altered intercellular communication, chronic inflammation, and dysbiosis (López-Otín et al., 2023).

The intricate interplay and cumulative impact of these features remain largely unexplored in the context of age-related diseases progression and healthspan in humans (Rolland et al., 2023; Addie et al., 2024; Fekete et al., 2024).

The middle-aging hypovascularity hypoxia hypothesis presents evidence linking menopause or andropause in middle-aging to decreased blood flow (hemodynamic) due to endogenous NO-mediated microvascular (hypovascularity) and oxygenation reduction (hypoxia) (Phua, 2023). A decrease in NO-mediated microvascular structure (microvasculature-hypovascularity) in tissues with hypoxia and hemodynamic factors reveal a complex biological dysregulation linked to the emergence of vascular aging and tissue hypoperfusion. Over time, vascular aging involves the deterioration in vascular structure and function and ultimately leads to cumulative tissue (cellular) damage in the heart, brain, kidney, and other organs (Climie et al., 2023).

The intricate relationships and cumulative effects of these aging hallmarks are best understood through the lens of Geroscience (Rolland et al., 2023; Addie et al., 2024; Fekete et al., 2024), and the methodologies from population health epidemiology (LaMorte, 2021). This integrated knowledge translation establishes connections across different levels of biological organization, highlighting causal relationships (LaMorte, 2021) and triangulating evidence (Hammerton and Munafò, 2021; Munafò et al., 2021) for a clearer understanding of unifying biological structures and functions entities (Herman et al., 2022). It provides a comprehensive and deeper understanding of how various biological (heterogeneity) processes are carried out across different systems-based levels (Landay et al., 2021; Falshaw et al., 2024) (systemic-cellular) during human aging.

## 2 Systemic level: sex hormones, nitric oxide deficiency and vascular aging hypovascularity

### 2.1 Sex hormones and nitric oxide deficiency

Both the prostate degeneration and middle-aging hypovascularity hypotheses indicate a decline in sex hormones and the NO-production during middle-aging (Phua, 2021; Phua, 2023). This period is marked by the cessation of estrogen production due to menopause (Cignarella et al., 2024) and a rise in serum testosterone deficiency (andropause) (Llukani et al., 2017; Erenpreiss et al., 2020; Kanabar et al., 2022). Advancing age is linked to lower overall NO-production in the body (Siervo et al., 2024), and the diminished bioavailability of NO in postmenopausal women is well established (Fredette et al., 2018; Somani et al., 2019).

In contrast, estrogen or menopause replacement therapy has been shown to significantly elevate plasma NO levels in postmenopausal women (Majmudar et al., 2000; Akhan et al., 2002). Similarly, testosterone replacement therapy results in a notable increase in NO-production (Campelo et al., 2012; Hotta et al., 2019; Gur et al., 2020; Akseh et al., 2021).

### 2.2 Nitric oxide deficiency and vascular aging hypovascularity

Testosterone or estrogen replacement therapy can effectively reverse the testosterone deprivation caused by orchiectomy in rats’ experiments with urethral hypovascularity (Yura et al., 2020; Gerbie et al., 2021). Hypogonadal status patients have been found to have decreased peri-urethral vascularity (Hofer et al., 2017).

Vascular aging, characterized by the many hypovascularity descriptions, is strongly linked to the progression of various age-related diseases, as highlighted by observational studies. Tissue hypoperfusion has been associated with Alzheimer’s disease (Salminen, 2021) and vascular cognitive impairment (Rajeev et al., 2023). Additionally, macro-micro-angiopathy is connected to diabetes (Madonna et al., 2017) and associated erectile dysfunction (Defeudis et al., 2022). Capillary rarefaction is related to sarcopenia (Hayashi, 2021; Jeon et al., 2021; Hendrickse et al., 2022) and has repercussions for renal and cardiovascular disease (Steegh et al., 2024). Decreased densities of microvessel and microvascular structures are indicative of diabetic myocardial injuries (Wang et al., 2024) and overall cardiovascular health issues (Wang et al., 2024).

In two 60-day studies, canine orchiectomy was found to reduce prostate vascularization (hypovascularity) (Angrimani et al., 2020), perfusion (hypoperfusion), and blood flow volume (Yoon et al., 2020). The findings observed offer a distinctive explanation of the interplay among endocrine system (specifically sex hormones), endogenous NO-production, and the vascular system at both the systemic regional and the local cellular tissue levels. The local cellular tissue vascular system (niches) is exemplified by the microvascular hemodynamic structure that facilitates perfusion function to the tissue. This establishes a cohesive unifying relationship between structure and function across all levels of biological organization (Herman et al., 2022).

This underscores the critical relationship between systemic regional systems and local cellular tissue vascular niches, which synergistically influence the advancement of aging-related chronic diseases. These interactions (systemic-cellular) result in a progressive pathobiological condition characterized by vascular aging hypovascularity and impaired tissue perfusion (hypoperfusion).

## 3 Cellular tissue level: vascular aging hypovascularity and tissue hypoperfusion-driven pathobiology

### 3.1 Systemic diseases and vascular aging hypovascularity

Microcirculatory dysfunction due to vascular aging is now recognized as a systemic issue (Feuer et al., 2022; Meariman et al., 2023; Wagner et al., 2023). Reduced blood flow plays a significant role in exacerbating various pathological conditions, such as angina pectoris, atherosclerosis, coronary artery and microvascular disease (Tracy et al., 2021). The topic of “Menopause and Your Heart” explores the metabolic syndrome, which heightens the risk of diabetes, hypertension, and weight gain (British Heart Foundation, 2024). The decline in estrogen production affects metabolism, raising the likelihood of obesity and diabetes (Cignarella et al., 2024). Additionally, capillary rarefaction is linked to obesity, metabolic disorders, and glucose homeostasis (Paavonsalo et al., 2020).

Estrogen plays a crucial role in maintaining the integrity of blood vessels in bone during both pregnancy and menopause (Rodrigues et al., 2022). Research indicates that the modulation of estrogen *via* estradiol stimulates the release of vasoactive substances, including NO and prostacyclin, while also promoting the production of angiotensin1-7 along the angiotensin axis (Novella et al., 2019). The production of angiotensin1-7 leads to a range of beneficial effects, such as vasodilation, reduced inflammation, prevention of fibrosis, inhibition of angiogenesis, and lower blood pressure (Touyz and Montezano, 2018), as well as improvements in glucose and lipid balance (Lelis et al., 2019).

Structural changes in small resistance arteries are regarded as the gold standard for evaluating hypertension, as opposed to the typical microvascular remodeling (Rizzoni et al., 2023). On the other hand, some researchers attribute hypertension to capillary and microvascular rarefaction (Mourad and Laville, 2006; Liang et al., 2019; le Noble et al., 2023). Hypertension associated with cancer therapies underscores the significant impact of factors such as decreased NO-generation, oxidative stress, endothelin-1, prostaglandins, endothelial dysfunction, heightened sympathetic activity, and microvascular rarefaction (Cohen et al., 2023).

Likewise, Chinese patients undergoing androgen deprivation therapy for prostate cancer face an increased risk of developing new instances of hypertension, diabetes, and hyperlipidemia (Wong et al., 2022). Similarly, it increases the risk of weight gain, emotional changes, cardiovascular disease, diabetes and osteoporosis (MacLennan et al., 2023).

### 3.2 Microvascular hemodynamic structure and hypoperfusion-driven pathobiology

Significantly, most human tissues are exposed to *in vivo* oxygen levels between 2% and 6% (bioavailability), commonly known as physoxia (McKeown, 2014) or physioxia (Adebayo and Nakshatri, 2022; Alva et al., 2022), due to blood flow circulation (Premont et al., 2020). The microcirculation within microvascular networks plays a crucial role in ensuring adequate tissue perfusion, delivering essential oxygen and nutrients, facilitating waste removal, and supporting immune functions. Such microcirculation is critical for cellular tissue function, regulation, and overall homeostasis (Guven et al., 2020; Munoz et al., 2020; Hsia, 2023; Satish and Tadi, 2023). However, the aging process is marked by a gradual decline in tissue perfusion (Wolters et al., 2017; Staffaroni et al., 2019).

**FIGURE 1:**
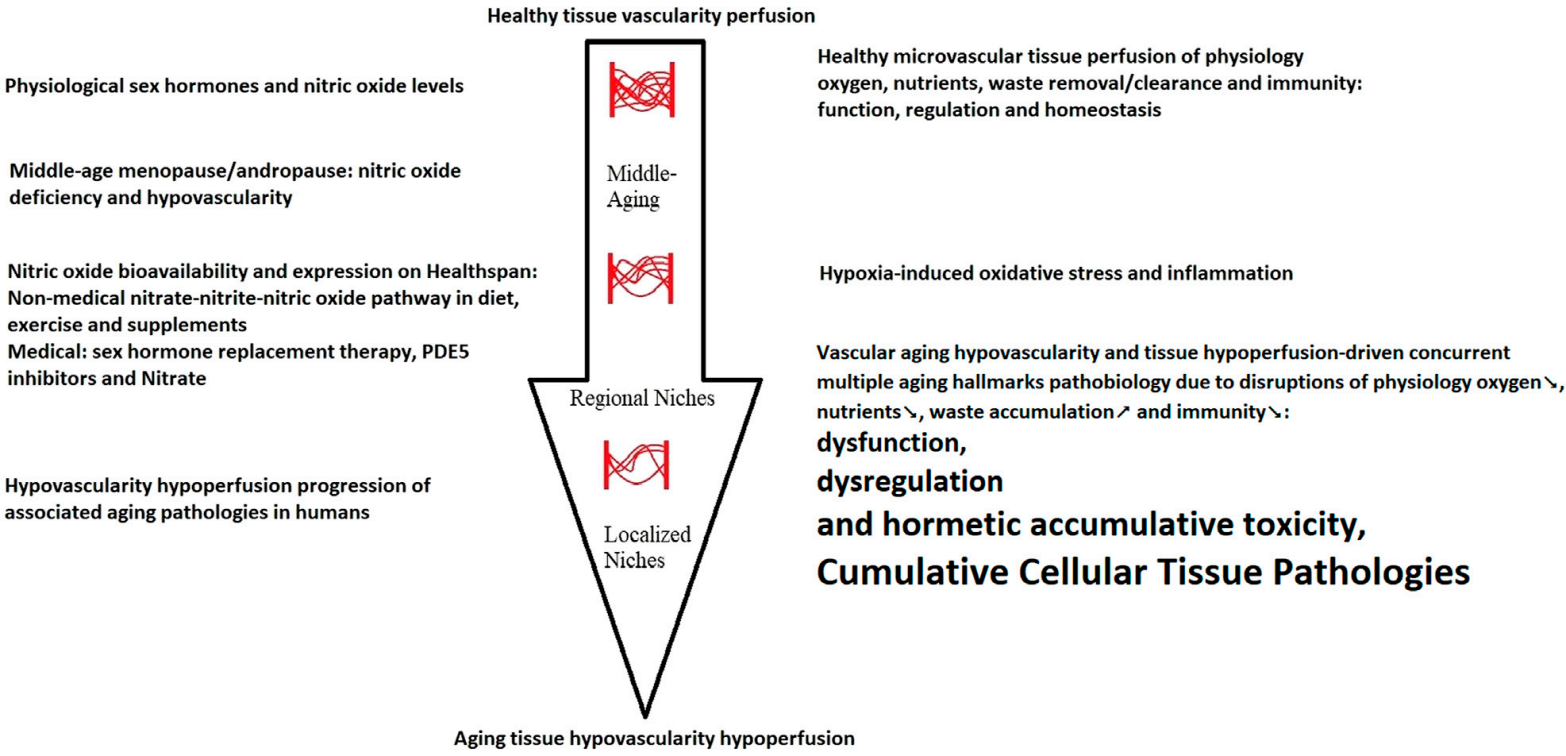
Aging nitric oxide deficiency and aging hypovascularity hypoperfusion.

This NO-mediated vascular aging hypovascularity and tissue hypoperfusion-driven impairment in delivering oxygen and nutrients, facilitating waste removal and immune function, triggers the concurrent activation of multiple aging hallmarks pathobiology mechanisms (Figure 1).

The few examples of individual hallmarks mentioned below illustrate the interactive presence of multiple aging hallmarks driven by hypovascularity and hypoperfusion pathobiology. A lack of oxygen delivery (hypoxia) leads to a decrease in metabolic rate due to the inability of mitochondria to perform oxidative phosphorylation (Wilson, 2017; Vercellino and Sazanov, 2022; Mao et al., 2024), resulting in a shift toward aerobic glycolysis, known as the Warburg effect (Pascale et al., 2020; Martins Pinto et al., 2023). Patients with suspected coronary microvascular disease exhibit unique microcirculatory resistance and myocardial metabolic profiles, both at rest and in response to physical activity (Noaman et al., 2023).

Impairments in nutrient supply involve the dysregulation of nutrient sensing, which impacts cellular metabolism, cellular senescence (proliferation), glands secretion (such as insulin), and promotes increased autophagy activity (González et al., 2020; Parmar et al., 2022; Huynh et al., 2023). Microvascular rarefaction may lead to an imbalance between perfusion and metabolic demand in metabolic syndrome, as local metabolic requirements are not adequately fulfilled due to insufficient nutrient and oxygen supply (Wong et al., 2022).

Furthermore, the microvascular hemodynamic vascular and lymphatic systems responsible for cellular waste removal become compromised, resulting in the buildup of wasteosomes (Riba et al., 2022). Corpora amylacea, which are starch-like bodies, are associated with tau in Alzheimer’s disease (Riba et al., 2023; Dallmeier et al., 2024) and are prevalent in cases of prostate enlargement (Sun and Bao, 2013; Badea et al., 2015; Ichimata et al., 2024). This blood stasis can clarify the hormetic-biphasic dose/concentration relationships of NO (Calabrese et al., 2023b) and defines the limits of lifespan (Calabrese et al., 2023a). The immunological aspect of pathobiology is characterized by a deterioration of immune function, commonly referred to as immunosenescence (Rodrigues et al., 2021; Sayed et al., 2021; Lee K.-A. et al., 2022).

In humans, vascular aging is characterized by a decline in microvasculature (hypovascularity) (Wei et al., 2017; Chua et al., 2024; Kellner et al., 2024), perfusion (hypoperfusion) (Lin et al., 2019; Liu et al., 2021), and hemodynamic (Kavroulakis et al., 2021; Leidhin et al., 2021; Roberts et al., 2023).

**FIGURE 2:**
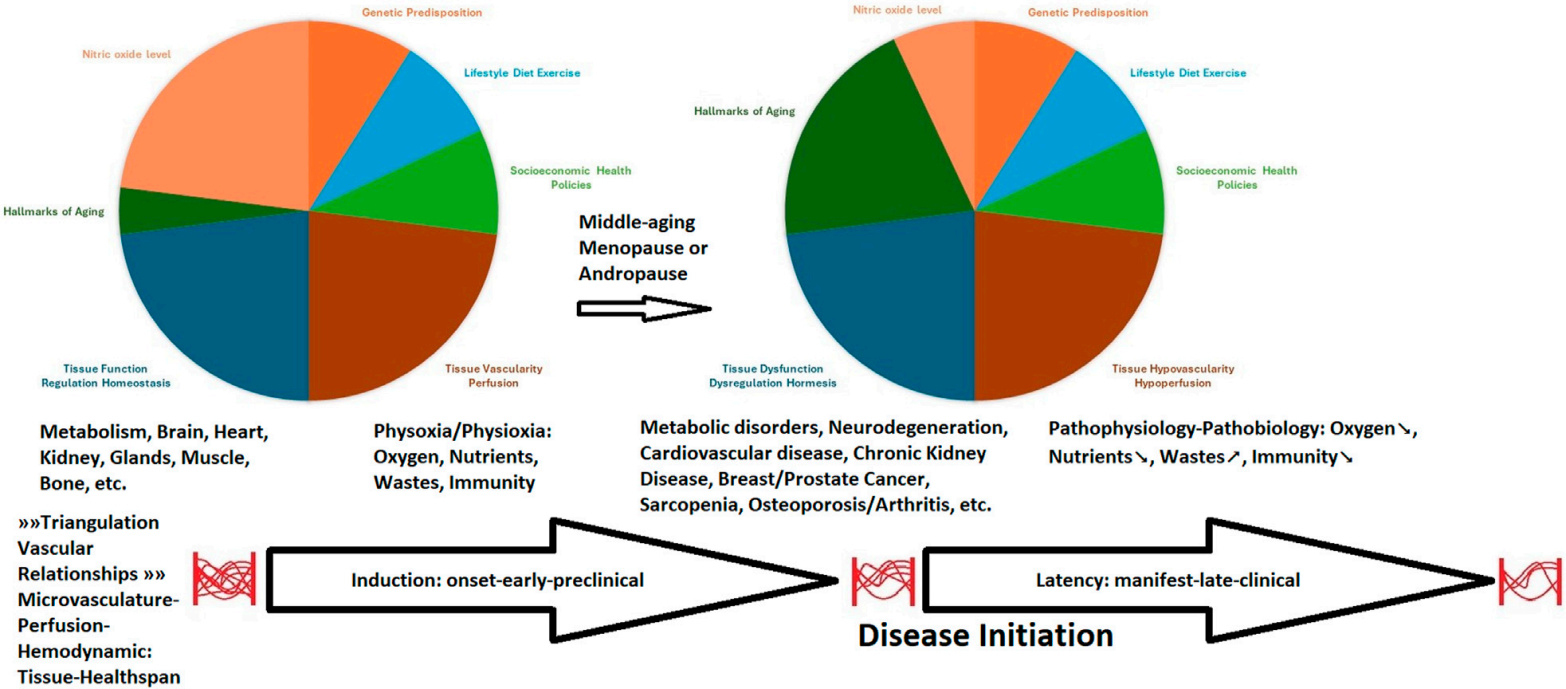
Triangulation causality in heterogeneity biological aging.

Applying the principles of population health epidemiology often leads to viewing components like microvasculature, perfusion, and hemodynamic purely as associations rather than as causal relationships (LaMorte, 2021). However, these components create a cohesive unifying framework linking structures and functions (Herman et al., 2022) within the context of the pathobiological effects driven by hypoperfusion, which can significantly influence cellular tissue healthspan. By triangulating related evidence (Hammerton and Munafò, 2021; Munafò et al., 2021), we can recognize these systemic endocrine-NO-vascular systems (Landay et al., 2021) as having causal links to cellular tissue microvascular hemodynamic structures and perfusion functions (microvasculature-perfusion-hemodynamic). This triangulation is particularly relevant in understanding the heterogeneity of biological aging and allows for more precise big picture causal inferences regarding the stages of disease progression, from onset to early preclinical induction phases and latency through to manifest and late clinical stages (LaMorte, 2021) (Figure 2).

### 3.3 Retinal microvasculature and aging biomarkers

Currently, there is a research gap in the validation of aging biomarkers that are suitable for clinical application (Bao et al., 2023; Moqri et al., 2024), particularly regarding their relationship with vascular aging and microvessel diseases (microvasculature) (Rizzoni et al., 2019; Mun et al., 2023).

The aging retinal microvasculature (Abay et al., 2022) presents a valuable opportunity for the translational validation of aging biomarkers. Numerous studies have investigated its structural and blood flow changes, revealing connections to vascular aging (Gómez-Sánchez et al., 2022), sex hormones (Malan et al., 2015; Aribas et al., 2022), and androgen deprivation therapy (Shin et al., 2020). Additionally, it shows correlations with chronic illnesses including dementia risk (Rebouças et al., 2023), chronic kidney disease/hypertension (Frost et al., 2021; Fursova et al., 2021), cardiovascular disease (Zhong et al., 2022; Kellner et al., 2024), diabetes (Kim et al., 2022; Marques et al., 2022), and hypertension (Zeng et al., 2022).

This necessitates the development of novel statistical analyses to determine a correlation coefficient index and ratio between retinal microvascular density and various aging biomarkers. These biomarkers include those related to oxidative stress, inflammation, oxygen levels, hypoxia-inducible factors, NO levels, amyloidosis (amyloid-beta), autophagy, epithelial-mesenchymal transition, sex hormones, and their associated symptoms. Such an approach would facilitate more accurate early prediction, intervention, screening, and monitoring, leading to a refined assessment of biological aging.

## 4 Nitric oxide bioavailability and healthspan

### 4.1 Nitric oxide: health and disease

In a healthy normative state, the NO-cyclic 3′-5′ guanosine monophosphate signaling pathway plays a critical role on smooth muscle tone, platelet activity, cardiac contractility, renal function and fluid balance, and cell growth (Mónica et al., 2016). NO has important roles in the regulation of kidney, cardiovascular and metabolic functions (Carlström, 2021). NO promotes and maintains vasodilation (Böger and Hannemann, 2020), angiogenesis (Zhang et al., 2023), and vascular function (Tejero et al., 2019; Costa et al., 2021; Loscalzo, 2024).

Conversely, decreasing levels of NO play a significant role in the development of hypertension (da Silva et al., 2021; Bryan, 2022) and are linked to the progression of age-related diseases (Bryan et al., 2023).

The contrasting effects of NO on health and disease highlight the potential of early NO-based therapeutic interventions to enhance healthspan and avert the emergence of cumulative health issues. Therefore, it is essential to investigate the genetic expression of NO and the adaptations to low-oxygen environments in high-altitude populations (Lee et al., 2020; Li et al., 2021; Pooja et al., 2021; Yu et al., 2022), as increased NO-production is a common reaction to hypoxic stress (Feelisch, 2018). Additionally, studies indicate that native highlanders experience lower mortality rates from cardiovascular diseases, diabetes, and cancer (Thiersch and Swenson, 2018; Wander et al., 2020; Burtscher et al., 2021).

Healthspan interventions primarily aim to counteract this age-related NO-deficiency (Sverdlov et al., 2014; Pourbagher-Shahri et al., 2021). However, the bioavailability and expression of different NO enhancers and signaling donors (Mintz et al., 2021; Andrabi et al., 2023) have not been extensively investigated in relation to early healthspan interventions.

### 4.2 Nitric oxide: sex hormones

Notably, research suggests that hormonal therapies may reduce all-cause mortality during menopause (Hodis and Mack, 2022; Qian et al., 2022; Qu et al., 2023; Xing et al., 2023) and andropause (Wang et al., 2021; Muehlenbein et al., 2022; Stallone and Oloyo, 2023; Yeap et al., 2024). This aligns with the enhanced NO-production associated with sex hormone replacement therapies. Furthermore, the timing hypothesis indicates that the beneficial effects of hormone therapy are most pronounced when treatment begins early (Mehta et al., 2019; Nudy et al., 2019).

### 4.3 Nitric oxide: phosphodiesterase-5 inhibitors

Phosphodiesterase-5 inhibitors (PDE5i) are integral to the NO-soluble guanylyl cyclase-cyclic guanosine 3′,5′-monophosphate signaling pathway (ElHady et al., 2023; Samidurai et al., 2023), aiding in the restoration of NO signaling (Lee M.-K. et al., 2022).

Regular use of PDE5i has been linked to a reduced risk of overall mortality and lower mortality rates among men with type2 diabetes (Anderson et al., 2016), as well as for men using it to treat erectile dysfunction, showing a decrease in major adverse cardiovascular events (Kloner et al., 2023). Long-term use of PDE5i, whether in men with or without pre-existing coronary artery disease, also correlates with a diminished risk of cardiovascular incidents and overall mortality (Soulaidopoulos et al., 2024).

Furthermore, the PDE5i tadalafil has been shown to significantly enhance cognitive performance (Otari et al., 2023). The initiation of PDE5i therapy in men with erectile dysfunction was associated with a lower risk of Alzheimer’s disease, especially among those who frequently received prescriptions (Adesuyan et al., 2024).

### 4.4 Nitric oxide: lifestyle choices

There is a clear connection between the production of endogenous NO and lifestyle choices that promote healthy aging. These choices include consuming dietary nitrates, participating in regular exercise, and using NO-enhancer supplements.

Consuming dietary nitrates enhances the nitrate-nitrite-nitric oxide pathway and has been shown to support healthy aging (Rocha, 2021) through the interactions between oral and gut microbiota and stomach acidity (Jones et al., 2021; Bryan et al., 2022; Bryan et al., 2023). Following a Mediterranean diet is linked to increase NO-production (Shannon et al., 2018; Mohajeri and Cicero, 2023). Long-term dietary nitrate treatment does not appear to affect lifespan in rats nor does it raise cancer risk; however, it may enhance vascular function, potentially extending healthspan (Carvalho et al., 2021).

Regular physical activity positively influences NO-generation (Tsukiyama et al., 2017; Bishop et al., 2023) and microvascular function (De Ciuceis et al., 2023; Hong and Park, 2024). Additionally, supplementation with L-arginine or L-citrulline has been shown to improve endogenous NO regulation and production (Rashid et al., 2020; Bahadoran et al., 2021; Kiani et al., 2022). Nonetheless, it is crucial to consider the potential long-term negative effects of L-arginine supplementation, especially within the elderly population (Huang et al., 2020).

### 4.5 Nitric oxide: medications

Nicorandil medication demonstrates cardioprotective and antianginal effects through its dual action as an ATP-dependent potassium channel agonist, which supports microvascular dilatation, and by promoting NO-mediated vasodilation in medium to large blood vessels (Goel et al., 2023). *In vitro* studies demonstrated that TOP-N53, a dual-action NO-donor and PDE5-inhibitor, can extend both lifespan and healthspan in *Caenorhabditis elegans* worms (Rudgalvyte et al., 2024).

## 5 Discussion

This article uses a multi-disciplinary systems-based (Landay et al., 2021) approach that integrates the principles of a unified framework of biological structures and functions that occur across all levels of biological organization (Herman et al., 2022). It also examines overarching big picture causal relationships (LaMorte, 2021) and triangulates related evidence (Hammerton and Munafò, 2021; Munafò et al., 2021).

Comprehending the processes of human aging, particularly the linked factors of declining sex hormones (endocrine), NO-deficiency, and reduced microvascular blood flow leading to hypoxia in middle age (Phua, 2023), create a timely opportunity for interventions aimed at improving NO levels, microvascular health, and overall hemodynamic tissue perfusion wellness.

Biological aging is characterized by a gradual decline in complex biological systems, including the endocrine system (especially sex hormones), the body’s production of NO, and the vascular system. This deterioration negatively affects local microvascular hemodynamic structures in cellular tissues, which are essential for adequate blood flow perfusion. The resulting hypovascularity and decreased blood flow, influenced by NO levels, disrupt the delivery of oxygen and nutrients to cells, hinder waste removal, and impair immune function. The cumulative aging pathologies in cellular tissues stem from the interconnected pathogenic effects of hypovascularity and the interactive multiple aging hallmarks pathobiology related to inadequate tissue perfusion (hypoperfusion) (Figures 1, 2).

Recognizing these interconnected interactions in human aging opens the door for future AI-driven strategies focused on aging-related changes in retinal microvasculature (Csipo et al., 2024) and aging biomarkers as comprehensive health indices for aging hallmarks. Such early interventions targeting the cellular tissue healthspan gap (Garmany et al., 2021), can promote healthy aging choices, lower medical expenses, and enhance the sustainability of healthcare systems.

## Data Availability

The original contributions presented in the study are included in the article/supplementary material, further inquiries can be directed to the corresponding author.
